# Autoimmune syndrome induced by adjuvants (ASIA) following urethral sling placement: A case report

**DOI:** 10.1016/j.crwh.2025.e00778

**Published:** 2025-12-24

**Authors:** Muhammed A.M. Hammad, Gustavo Gryzinski, Evelyn Minji Pak, Alexander Bell, Gamal Ghoniem

**Affiliations:** a University of California, Irvine, USA; b Department of Urology, University of California, Irvine, USA

**Keywords:** Autoimmune syndrome induced by adjuvants (ASIA), Polypropylene mesh, Mid-urethral sling, Systemic autoimmune reaction, Mesh explantation, Gynecologic surgery complications

## Abstract

A 39-year-old woman developed debilitating systemic symptoms following the placement of a synthetic mid-urethral sling for stress urinary incontinence. Previously in good health, she experienced sepsis and adrenal crisis immediately postoperatively, followed by persistent symptoms including fatigue, postural orthostatic tachycardia syndrome (POTS), neuropathic pain, cognitive dysfunction, autonomic instability, and recurrent infections. Despite extensive multispecialty evaluations, no definitive diagnosis was initially reached. Laboratory testing later revealed positive ANA and mild immune dysregulation. Given her complex autoimmune history, strong family predisposition, and the temporal association with mesh implantation, her presentation was suspected to be consistent with autoimmune syndrome induced by adjuvants (ASIA). She elected to undergo surgical removal of the synthetic sling with placement of an autologous rectus fascia pubovaginal sling. Postoperatively, the patient experienced significant resolution of her symptoms, restored bladder function, and improved quality of life. This case demonstrates a temporal association consistent with ASIA, with complete symptom resolution by 3 months post-explant and sustained well-being at 1 year.

## Highlights

• 39-year-old woman developed autoimmune-like symptoms following placement of transobturator polypropylene sling.
• Autoimmune Syndrome Induced by Adjuvants (ASIA), supported by temporal association between mesh implantation &symptoms
• Surgical explantation with autologous rectus fascia resulted in near-complete resolution of symptoms.
• Need for multidisciplinary evaluation in unexplained systemic illness following sling.
• Potential association between synthetic mesh & immune activation, reinforcing individualized management.

## 1 Introduction

Synthetic mid-urethral slings (MUS) have long been considered the gold standard for the surgical management of stress urinary incontinence due to their high success rates and minimally invasive approach. In extremely rare cases, synthetic materials such as polypropylene mesh have been associated with triggering a constellation of systemic symptoms consistent with autoimmune syndrome induced by adjuvants (ASIA), a condition characterized by immune dysregulation in genetically susceptible individuals. ASIA is a complex syndrome resulting from the interaction of genetic predisposition and environmental factors with adjuvants, through modulation of receptors, such as TLR, NLR and CLR, triggering aberrant immune responses. First described by Shoenfeld and Agmon-Levin in 2011, ASIA encompasses a range of symptoms including fatigue, arthralgia, and cognitive dysfunction following exposure to adjuvant materials. Since 2011, more than 4479 cases of ASIA syndrome have been reported. Approximately 92.7 % of these occurred in women and were mainly associated with adjuvants in HPV and hepatitis B (HBV) vaccines. In a systematic review by Jara et al., severe cases accounted for 6.8 % of the total, with a mortality rate of 0.24 % [1]. Although the pathophysiology of ASIA remains incompletely understood, increasing recognition of mesh-related autoimmune phenomena has impelled reevaluation of synthetic slings in select patients with complex autoimmune backgrounds.

## 2 Case Presentation

A 39-year-old woman presented for consultation in a tertiary care center due to persistent systemic symptoms following the placement of a urethral sling. The patient had stable, well-controlled autoimmune endocrinopathies (Hashimoto's thyroiditis and Addison's disease) and was otherwise healthy and active prior to sling placement. The index sling was a retropubic polypropylene mid-urethral sling. It remained in situ for approximately 18 months prior to explantation. Following the procedure, she developed bacteremia with sepsis and adrenal crisis, requiring hospitalization for one week. Upon discharge, she continued to experience worsening symptoms, prompting extensive evaluations by multiple specialists. Her symptoms included severe fatigue, postural orthostatic tachycardia syndrome (POTS), recurrent infections, temperature dysregulation, cognitive dysfunction, neuropathic pain, and autonomic disturbances. These symptoms were exacerbated by stress, despite normal cortisol levels. Additional complaints included pleuritic chest pain, balance disturbances, and profound brain fog with dissociation. Given the timeline of symptom onset immediately after surgery, concerns were raised about the potential for an autoimmune syndrome triggered by the synthetic mesh of the urethral sling.

### 2.1 Initial Clinical Assessment and Workup

The patient underwent several laboratory and imaging evaluations. ANA testing was initially negative at first follow-up but later returned positive at 1:160 with a speckled pattern after few months. Additional serologies, including ANCA, cardiolipin, lupus anticoagulant, and a comprehensive myositis panel, were negative. Paraneoplastic screening and evaluation for POEMS syndrome were unremarkable. VEGF levels were noted to be low, though this finding did not meet the criteria for POEMS syndrome. IL-13 was mildly elevated, suggesting immune dysregulation. Imaging showed no evidence of cardiopulmonary pathology.

The patient's past medical history was significant for Hashimoto's thyroiditis, thyroid cancer, Addison's disease, polycystic ovarian syndrome (PCOS), panic disorder, and post-partum depression. A strong family history of autoimmune disease was noted, with both parents diagnosed with Hashimoto's thyroiditis and a paternal grandmother with rheumatoid arthritis.

Notably, ASIA has no specific histopathologic criteria, and chronic foreign-body reaction alone does not exclude the diagnosis. The absence of acute inflammation is expected with many polypropylene explants and is consistent with reports in the literature. The atypical constellation of symptoms at her presentation was consistent with ASIA, a condition triggered by immune system hyperactivation in response to foreign material. Given that her symptoms began immediately after urethral sling placement, this was considered the most plausible etiology.

### 2.2 Surgical Management and Postoperative Course

Due to persistent systemic symptoms consistent with ASIA, the patient elected to undergo surgical removal of the synthetic sling. The procedure was performed at an academic institution under general anesthesia. The surgery team prepared the patient with chlorhexidine/alcohol and used intraoperative gentamicin irrigation (240 mg in 2 L normal saline), also infiltrating incision sites with bupivacaine. Vaginal packing with metronidazole cream was used. A combined vaginal and abdominal approach was used. Excision included removal of both retropubic arms and the entire suburethral segment, ensuring no residual polypropylene material. A concurrent urethrolysis and placement of an autologous pubovaginal sling using rectus fascia was performed. [2] The rectus fascia was harvested through a Pfannenstiel incision and fashioned into an approximately 8–10 cm × 1.5–2 cm strip and suspended using 2–0 PDS (polydioxanone) absorbable sutures at each end. The suture ends were brought to the abdominal incision through the retropubic space using Stamey's needle passers. Cystoscopy confirmed no bladder or urethral injuries. There were no intraoperative complications. The histopathologic examination of both right and left sling segments revealed polypropylene mesh with adherent fibrous tissue, and no acute inflammation or infection, which was consistent with a chronic foreign-body reaction.

At her initial postoperative visit, the patient reported near-complete resolution of systemic and local symptoms, including cognitive dysfunction, neuropathic pain, temperature dysregulation, and fatigue. She voided spontaneously to completion and denied incontinence, dyspareunia, or urinary tract infection. On physical examination, her Pfannenstiel incision was well healed with no evidence of infection, tenderness, or seroma. The vaginal walls were well supported, with normal anterior and posterior compartments (POP-Q stage 0). A post-void residual urine volume of 8 mL confirmed satisfactory bladder emptying.

Approximately 20 days postoperatively, she presented twice to outside emergency departments for urinary retention. Computed tomography (CT) revealed a 6 × 2 cm retropubic seroma and a urinary tract infection, both managed successfully with oral ciprofloxacin. She subsequently voided normally with a post-void residual of 50 mL, remained afebrile, and experienced no wound complications. The episode resolved without further sequelae. Postoperative laboratory evaluation demonstrated no systemic inflammatory response beyond a transient urinary tract infection, with normal leukocyte count and inflammatory markers thereafter.

At her 3-month follow-up, the patient continued to report complete resolution of systemic and urinary symptoms. She denied urgency, incontinence, recurrent infections, or fatigue and had no signs of autonomic dysfunction. Physical findings remained unchanged, and she expressed high satisfaction with the surgical outcome. She remained asymptomatic and voluntarily canceled her one-year follow-up appointment, reporting sustained well-being and no recurrence of urinary or systemic symptoms.

## 3 Discussion

ASIA is a rare clinical entity characterized by fatigue, musculoskeletal pain, neurological impairment, and immune dysfunction following exposure to exogenous materials such as silicone, vaccines, or synthetic implants. The exact pathophysiology is unclear but is thought to involve adjuvant-driven immune activation leading to systemic inflammatory and autoimmune phenomena.

ASIA has been linked to various foreign materials, including polypropylene mesh implants used in urogynecologic and hernia surgeries. While rare, cases of ASIA following urethral sling placement have been reported, highlighting the need for clinician awareness when evaluating post-surgical immune-related complications. Reports of ASIA induced from polypropylene implants, including cases involving hernia repairs, showed that mesh removal led to symptom improvement [2].

In nonhuman primates, sacrocolpopexy using polypropylene vaginal mesh also showed a correlation between mesh burden and lipid oxidation [3]. In rats, while most showed no effects, a subset developed elevated hs-CRP, immune complications, and local tissue complications [4]. These findings suggest that individual variability including genetic predispositions and environmental factors may influence susceptibility to ASIA.

In this case, the immediate onset of symptoms postoperatively, the absence of a prior autoimmune diagnosis, and the marked symptom resolution following sling removal represents a temporal association consistent with ASIA criteria rather than a definitive causal relationship. The patient's systemic symptoms, including POTS, cognitive dysfunction, and autonomic disturbances, likely represent a dysregulated immune response to the synthetic mesh. Despite her positive ANA, she did not meet criteria for a specific autoimmune disease such as systemic lupus erythematosus (SLE) or mixed connective tissue disease. This underscores the importance of recognizing ASIA as a differential diagnosis in patients with unexplained systemic symptoms following implantable device placement.

Other materials, such as silicone implants, metal orthopedic implants, Essure devices, and metal dental crowns have also been linked to ASIA [[5], [6], [7], [8]], indicating a wider range in materials that trigger ASIA. (Table 1) The postoperative urinary tract infection and small retropubic seroma were localized, without systemic sepsis or bacteremia. Pathologic findings confirmed the absence of infection or acute inflammation, supporting a non-infectious, immune-mediated mechanism.

**Table 1:** Summary of implants in the literature leading to ASIA or ASIA-like symptoms.

Study	Model/Population	Implant Material	Main Findings	ASIA-Relevant Domains	Implications
Fadaee et al., 2023 [2]	Clinical case series (hernia patients)	Polypropylene mesh	Symptom resolution after mesh removal in patients with systemic inflammatory symptoms	Neurologic, immunologic, musculoskeletal	Supports link between mesh and systemic illness (mesh implant illness/ASIA)
Artsen et al., 2025 [3]	Nonhuman primates (sacrocolpopexy model)	Polypropylene mesh	Correlation between mesh burden and lipid oxidation in vaginal tissue	Immunologic	Supports link between mesh burden and oxidative stress contributing to ASIA
Eisenach et al., 2025 [4]	Rat model	Polypropylene mesh	Subgroup developed elevated hs-CRP, immune complications, and local tissue issues	Immunologic, inflammatory	Suggests genetic and environmental conditions affect susceptibility to ASIA
Cohen Tervaert et al., 2023 [5]	Multiple implant types (review of ASIA cases)	Various adjuvants (incl. Mesh, silicone, metal)	Overview of ASIA presentations and adjuvant triggers	Neurologic, immunologic, musculoskeletal, vascular	Highlights major adjuvant triggers of ASIA and need for physician awareness
Tervaert et al., 2024 [6]	Breast implant patients	Silicone	Breast implant illness linked to ASIA symptoms	Neurologic, immunologic	Supports association of silicone breast implants with ASIA
Kagan et al., 2020 [7]	38-year-old female with porcelain-fused-to-metal crown (PFM)	Dental PFM crown Metal component: chrome and cobalt alloy	Dental implant induced ASIA-like symptoms. Removal led to recovery	Neurologic, immunologic	Suggests even smaller scale medical implants like dental implants can trigger ASIA in susceptible individuals
Schiff et al., 2021 [8]	2 patient case studies: One 75-year-old female who underwent surgery of her left radius bone One 65-year-old female who underwent open reduction internal fixation surgery of right distal ulnar bone fracture	Orthopedic metal hardware (incl. DePuy Synthes titanium plates, screws)	Both patients developed ASIA-like symptoms after metal implant fixation procedures. Removal of hardware led to clinical improvement	Neurologic, immunologic, musculoskeletal	Supports association of orthopedic metal implants with ASIA

Included reports extend beyond urogynecologic implants to illustrate the broad range of adjuvant-related ASIA presentations**.**

A large, matched cohort study by Chughtai et al. found no increased incidence of autoimmune disease among women with vaginal mesh compared with non-mesh procedures, suggesting that ASIA is an exceptionally rare phenomenon in genetically predisposed individuals [9]. Additionally, a National Health Service (NHS) study found no statistically significant increase in the long-term risk of autoimmune disease, fibromyalgia, or myalgic encephalomyelitis in women who had synthetic mesh sling procedures compared with those who had non-mesh incontinence surgery [10]. Similarly, a recent large national cohort study in England by Muller et al. found no increase in long-term incidence of autoimmune disease among women receiving a synthetic mid-urethral mesh sling compared with non-mesh surgery (adjusted HR 0.89, 95 % CI 0.79–1.01) [11].

As large-scale epidemiologic studies demonstrate no increased incidence of autoimmune disease in women receiving mid-urethral slings, this case represents an idiosyncratic reaction in a genetically predisposed individual, rather than evidence of a generalized autoimmune risk associated with polypropylene slings. Autologous slings remain as feasible alternative [12].

Ultimately, this case reinforces the importance of considering ASIA in patients with unexplained symptoms after implanting polypropylene mesh, especially those with a history of autoimmune disorder (which may be revealed only on prompting). The patient remained symptom-free through one year after explantation, reinforcing the durability of clinical remission and supporting a true de-challenge effect. Early recognition and removal of adjuvants is optimal to manage ASIA symptoms and improve patient outcomes in these cases.

## 4 Conclusion

This case illustrates an erratic but clinically significant presentation of ASIA following urethral sling placement, in a person with underlying autoimmune susceptibility. The patient experienced profound systemic symptoms resistant to medical therapy, which significantly impacted her quality of life. Surgical removal of the synthetic sling and replacement with an autologous rectus fascia pubovaginal sling led to near-complete resolution of symptoms, demonstrating a temporal association between the implant and immune dysregulation, with complete and durable symptom resolution following mesh explantation. The case highlights the need for heightened awareness of ASIA among urologists, rheumatologists, and primary care providers when evaluating patients with post-implantation autoimmune or autonomic dysfunction.

## Contributors

Muhammed A. M. Hammad contributed to conception of the case report, acquiring and interpreting the data, drafting the manuscript, undertaking the literature review and revising the article critically for important intellectual content.

Gustavo Gryzinski contributed to patient care, drafting the manuscript and revising the article critically for important content.

Evelyn Minji Pak contributed to undertaking the literature review and revising the article critically for important intellectual content.

Alexander Bell contributed to patient care, conception of the case report and interpreting the data.

Gamal Ghoniem contributed to patient care, the conception of the case report, acquiring the data, and revising the article critically for important intellectual content.

All authors approved the final submitted manuscript.

## Patient consent

Written informed consent was obtained from the patient for publication of the case report.

## Provenance and peer review

This article was not commissioned and was peer reviewed.

## Funding

No funding from an external source supported the publication of this case report.

## Declaration of competing interest

The authors declare that they have no competing interest regarding the publication of this case report.
